# Knowledge, attitudes and practices regarding rabies and exposure to bats in two rural communities in Guatemala

**DOI:** 10.1186/s13104-014-0955-1

**Published:** 2015-01-10

**Authors:** David Moran, Patricia Juliao, Danilo Alvarez, Kim A Lindblade, James A Ellison, Amy T Gilbert, Brett Petersen, Charles Rupprecht, Sergio Recuenco

**Affiliations:** Universidad del Valle de Guatemala, Guatemala City, Guatemala; Centers for Disease Control and Prevention Regional Office for Central America and Panama, Guatemala City, Guatemala; Global Disease Detection Branch, Center for Global Health, Centers for Disease Control and Prevention, Atlanta, GA USA; USDA/APHIS/WS/National Wildlife Research Center, Fort Collins, CO USA; Ross University School of Veterinary Medicine, Basseterre, St. Kitts West Indie; Division of High-Consequence Pathogens and Pathology, Centers for Disease Control and Prevention, Atlanta, GA USA

**Keywords:** Rabies, Bat bite, Health practices, Post-exposure prophylaxis, Guatemala, Rabies prevention, Vampire bat

## Abstract

**Background:**

Rabies is a fatal encephalitis caused by rabies virus, of the genus *Lyssavirus*. The principal reservoir for rabies in Latin America is the common vampire bat *(Desmodus rotundus),* which feeds routinely on the blood of cattle, and when livestock are scarce, may prey on other mammals, including humans. Although rabies is endemic in common vampire bat populations in Guatemala, there is limited research on the extent of exposure to bats among human populations living near bat refuges.

**Results:**

A random sample of 270 of 473 households (57%) in two communities located within 2 Km of a known bat roost was selected and one adult from each household was interviewed. Exposure to bats (bites, scratches or bare skin contact) was reported by 96 (6%) of the 1,721 residents among the selected households. Of those exposed, 40% received rabies post-exposure prophylaxis. Four percent of household respondents reported that they would seek rabies post exposure prophylaxis if they were bitten by a bat.

**Conclusions:**

These findings demonstrate that exposure to bats in communities near bat roosts is common but recognition of the potential for rabies transmission from bats is low. There is a need for educational outreach to raise awareness of bat-associated rabies, prevent exposures to bats and ensure appropriate health-seeking behaviours for bat-inflicted wounds, particularly among communities living near bat roosts in Guatemala.

## Background

Rabies, an acute fatal encephalitis that affects all mammals, including humans, is a worldwide zoonotic disease caused by *rabies virus* (RABV) and, rarely, other members of the genus *Lyssavirus* [1]. The common vampire bat, *Desmodus rotundus*, is the principal reservoir of RABV in Latin America [2-4]. Vampire-bat rabies has been documented from Mexico to Argentina [5], and has been reported in Latin America since the early 20th century [6]. Although the disease was reported earlier among animals in Brazil, the first human rabies case acquired from vampire bats was reported on the island of Trinidad during the early 1930s [7]. Since then, human depredation by vampire bats and human rabies acquired from bats and has continued to be documented throughout Latin America [8-11].

Despite diminishing numbers of human rabies cases in Latin America due to control measures in canine populations, bat-associated rabies cases in humans and cattle have increased in many of these countries during the last decade [12-15]. Circulation of RABV among vampire bats throughout their geographic range has been demonstrated by extensive reports of vampire bat-associated RABV infections in bats, humans, and cattle throughout Latin America [16-19].

Although rabies transmitted by vampire bats is responsible for a greater burden of cases, human rabies associated with non-haematophagous bats also occurs in Latin America [4,6]. A bite is considered the most likely route of transmission of bat RABV to humans, even among individuals with no documented bite history. Different bat species vary in their degree of human interaction and peridomestic behaviour, and their reclusive habits can create rather unusual exposure circumstances. Some people may not recognize that they have received a bat bite or that there is a risk of rabies: compared to carnivores, bats are smaller-bodied mammals, and the wounds they inflict may not be appreciated as a potential rabies exposure, especially compared to bites from larger animals such as dogs [6,20,21].

In Guatemala, bats have been associated with several important zoonotic pathogens besides RABV [22-24]. Although the Guatemalan Ministry of Public Health and Social Assistance (MSPAS) registers animal bites to humans in its epidemiological surveillance system, there is limited reporting of bat bites or other bat exposures among the population. To better understand the potential for human exposure to bat-associated rabies, and the actions taken by people after being exposed to bats, we conducted a cross-sectional survey among the human population living next to two bat roosts in southern Guatemala to assess exposure to bats, knowledge of rabies, attitudes towards bats, practices related to bat avoidance and health-seeking behaviour following bat exposures.

## Methods

### Study sites

Two rural communities in southern Guatemala, with bat roosts identified by local and national bat experts, were selected for the study: Microparcelamiento El Naranjo (Municipio of Santa Lucia Cotzumalguapa, Department of Escuintla; N14°20′41.5″/W91°0.013′44″), and Violetas del Jobo (Municipio of Taxisco, Department of Santa Rosa; N14°04′30.9″/W90°34′22.5″). Both communities have an agrarian economy based on sugar cane and rubber plantations, as well as cattle ranching.

### Study design and sampling

We used aerial photographs (Guatemalan Ministry of Agriculture and Animal Health 2006, scale 1:20,000) and geostatistical software (Hawths Tools® extension in ArcGIS®) to randomly select 150 households located within two kilometres of a bat cave from each of the study communities. Within each selected household, one adult ≥18 years of age was selected to respond to both a household level survey and an individual survey. The household level survey requested information for all household members, irrespective of age.

We chose a convenience sample size of 150 households per community because this number could be surveyed in one week. With a sample size of 300, the confidence limits around the estimated proportion of respondents with bat exposure would be +/− two percentage points assuming bat exposure was ≤10%.

## Informed consent

The study was approved by the ethics committee of the Universidad del Valle de Guatemala (UVG), Guatemala City, Guatemala and the institutional review board of the Centres for Disease Control and Prevention (CDC), Atlanta, GA, USA (protocol 5757.0). Written or thumbprint records of informed consent were obtained from all respondents prior to enrolment into the study.

### Data collection

The household level survey was representative of all household members, although questions were asked of a single respondent. The questionnaire included sections to capture information on demographics, exposure to bats, risk factors for bat exposures and history of rabies vaccination. Bat exposure was defined as having been bitten or scratched by a bat, or touching a bat with bare hands at any time in the past. Household respondents who reported receiving rabies vaccination were asked to indicate whether it was in direct response to an animal exposure, such as post-exposure prophylaxis (PEP), or as pre-exposure immunization.

Household respondents were asked evaluated using an individual survey requesting information on their knowledge, attitudes and practices related to bats and rabies. To assess rabies-related knowledge, participants were asked to self-rate their understanding of rabies, explain how humans acquire rabies, and identify animal sources of the disease. Each knowledge question was evaluated independently, and the validity of a participant’s self-reported knowledge level was not verified using other responses. Participants were also asked to describe the severity of rabies. Only responses that emphasized death were considered evidence that a respondent had knowledge that rabies is fatal.

To assess potential health-seeking practices following bat exposures, participants were asked hypothetical questions about actions they would take if they were bitten or scratched by a bat. Responses to this open-ended question were compared to a similar question asked later about actions a person should take following a bite from a potentially rabid animal. Questions that were specifically asked about bats preceded all questions about rabies to minimize reporting bias, and whenever feasible, participants were asked open-ended questions to minimize the interviewer’s influence on responses. After data collection was completed, answers to open-ended questions were grouped and coded for analysis.

Responses to the questionnaire were recorded using a personal digital assistant equipped with a global positioning system. Proprietary survey software was used for questionnaire development (Questionnaire Mobile, Informatics team, Emerging Infections Unit, Centre for Health Studies, Universidad del Valle de Guatemala).

### Statistical analysis

Responses were summarized using descriptive statistics. Respondent data were stratified by community and history of bat exposure, respectively. Variables of interest were compared between exposed and non-exposed persons using odds ratios (OR) with 95% confidence intervals (CI) and p-values were calculated with the chi square test or Fisher’s exact test as appropriate. Continuous variables were analyzed using t-tests. For all tests, the level of significance was evaluated at α = 0.05. Statistical analysis was performed using package Rcmdr® (John Fox, and collaborators 2011) for the R® software, version 2.11.1 (R Foundation for Statistical Computing, Vienna, Austria).

## Results

### Household survey

Using aerial photographs, 300 households were randomly selected from 473 households located within two kilometres of a bat roost, and 270 (90%) households participated in the survey. The total number of residents among enrolled households was 1,721. The mean age of household respondents was 41 years, 73% of respondents were female, and 9% had completed primary school (Table 1). Domestic animals were owned by 83% of households, and 22% of households reported a history of at least one owned animal bitten by bats. The rabies vaccination rate among animals owned by the household was high: 57% reported vaccinating at least one of their animals against rabies. One-third of households had windows or doors that prevented bat entry and 11% of respondents reported using a mosquito net to prevent bat bites. More than a quarter (28%) of households reported at least one resident with a history of bat exposure. Among the 1721 residents in the enrolled households, 77 (5%) respondents reported a history of bat exposure at any time in their life, with 41/77 (53%) having been bitten (data not shown).

**Table 1:** **Demographics and risk factors for bat exposure among households near a bat roost in Guatemala**

**Variables**	**El Naranjo**	**El Jobo**	**Total**
	**N = 124**	**N = 146**	**N = 270**
	n (%)*	**n (%)**	**n (%)**
**Household characteristics**			
Household size (mean, range)	6.5 (1 – 18)	5.0 (1 – 15)	5.7 (1–18)
Age of household respondent (mean, range)	40.7 (18–80)	40.5 (18–83)	40.5 (18–83)
Female household respondent	87 (70)	110(75)	197 (73)
Household respondent completed primary school	11 (9)	13 (9)	24 (9)
**Household level risk factors for bat exposure**			
Windows/doors prevent bat entry	41 (33)	48 (32)	89 (33)
Pets or livestock	98 (79)	127(87)	225 (83)
Pets or livestock bitten by bats	15 (12)	33 (23)	48 (22)
≥1 livestock/pet vaccinated against rabies	59 (48)	95 (65)	154 (57)
Mosquito net used to prevent bat entry	6 (5)	24 (16)	30 (11)
Measures taken to avoid animals being bitten by bats	43 (35)	50 (34)	93 (34)
Any bat exposure in a household member^§^	45 (36)	32 (22)	77 (28)

^§^Bat exposure defined as a bite, scratch or contact with unprotected skin.

*Percentages are proportions of non-missing data.

### Knowledge, attitudes and practices related to bats and rabies

Several factors were associated with exposure to bats. A lower risk for bat exposure was observed among persons less than or equal to 46 years (OR 0.46, 95% CI 0.25 – 0.81), and females (OR 0.38, 95% CI 0.21-0.70) (Table 2).

**Table 2:** **Risk factors associated with exposure to bats among persons living near bat refuges in Guatemala**

**Category**	**Subcategory**	**Exposed to bats (N = 77)**	**Non-exposed to bats (N = 193)**	**Total (N = 270)**	**P-value**	**Odds ratio (95% CI)**
		**n (%)**	**n (%)**	**n (%)**		
Demographics	Age ≤ 46 years^§^	42 (55 )	140 (73)	182 (67)	0.006	0.46 (0.25 -0.81)
	Female	45 (58)	152 (79)	197(73)	0.001	0.38 (0.21 -0.70)
	Completed primary school	14(18)	51 (26)	65 (24)	0.21	0.62 (0.29-1.23)
Rabies knowledge	Basic or no rabies knowledge	77(100)	192 (99)	269 (99)	1	0.80 (0.03- 20.7)
	Animal bites as mechanism of transmission	56(73)	136(70 )	192 (71)	0.76	1.11 (0.60-2.12)
	Rabies perceived as severe	66 (86)	137(71)	203 (75)	0.01	2.44 (1.17-5.52)
	Bats a source of rabies	9(12)	17(9)	26(10)	0.50	1.36 (0.51-3.43)
	Dogs a source of rabies	72(94)	176(91)	284(92)	0.62	1.38 (0.47-5)
Bat contact activities	More than 5 years living/working near bat roost	42(55)	62(32)	104(39)	0.0009	2.53 (1.42-4.51)
	Hunting bats (to kill, not eat)	0 (0)	2(1)	2(1)	1	0.6 (0.03-13.9)
	Agricultural occupation	13(17)	13(7)	26(10)	0.02	2.80 (1.13-6.94)
	Being inside a bat cave	49(64)	65(34)	114(42)	0.000001	3.43(1.91-6.23)
Theoretical actions after bat bite	Wash with soap and water	9(12)	12(6)	21(8)	0.13	1.99 (0.70-5.41)
	Seek medical care	40(52)	112(58)	152(56)	0.41	0.78 (0.44-1.38)
	Seek rabies post exposure prophylaxis	3(4)	9(5)	12(4)	1	0.83 (0.14-3.44)
	Don’t know or nothing	13(17)	28(15)	41(15)	0.70	1.19 (0.53-2.56)
	Other (alcohol/lime juice)	6(8)	13(7)	19(7)	0.79	1.17 (0.35-3.45)
Theoretical action after bite from rabid animal	Wash with soap and water	2(3)	7(4)	9(3)	1	0.70 (0.07-3.84)
	Seek medical care	56(73)	127(66)	183(68)	0.31	1.38 (0.75-2.62)
	Seek rabies post exposure prophylaxis	14(18)	35(18)	49(18)	1	1.03 (0.46-2.06)
	Don’t know or nothing	2(3)	15(8)	17(6)	0.16	0.32 (0.03-1.41)
	Other (alcohol/lime juice)	0 (0)	0(0)	0(0)	1	2.5(0.05-127.9)
History of vaccination against rabies	Post-exposure prophylaxis	12(16)	3(2)	15(6)	0.000003	11.57 (2.99-65.9)
	Pre-exposure immunization	5(6)	9(5)	14(5)	0.55	(0.36-4.90)
History of animal bite?	Bat	7(9)	0(0)	7(3)	0.0001	38.5(2.15-687.3)
	Dog	5 (7)	5(3)	10(4)	0.15	2.6(0.58-11.66)

^§^Mean age of exposed.

Regarding knowledge, attitudes and practices, 71% (192/270) of the participants indicated animal bites as a mechanism of RABV transmission. When asked to identify which animals can transmit rabies, 10% (26/270) of participants identified bats. Dogs were identified by 92% (248/270) of respondents, cats were identified by 27% (72/270) of respondents, and other mammals (e.g. rodents, swine and large domestic animals) were identified by 17% (46/270) of respondents, as animals that can transmit RABV. Regarding whether bats host other zoonoses, 10% of participants (27/270) answered affirmatively. Concerning the respondent self-assessment of rabies knowledge, 96% (258/270) reported having little or no knowledge of rabies, yet 71% (193/270) of respondent associated animal bites with transmission of RABV. Seventy-five percent (203/270) of respondents described rabies as severe, with a higher proportion among exposed persons (OR 2.44, 95% CI 1.17 – 5.52) (Table 2).

Eight percent (21/270) of the respondents reported that they would wash the injuries using soap and water if they were bitten or scratched by a bat, and 3% (9/270) reported the same wound treatment if they were bitten by an animal suspected to be rabid. Fifty-six percent (152/270) of respondents reported that they would seek medical care for a bat bite or scratch, while a higher proportion (68%, 183/270) advocated this action if the bite came from a potentially rabid animal. Only 7% (19/270) of respondents indicated they would apply alcohol and/or lime juice after being bitten by a bat, but no respondent reported such action if they were bitten by a suspected rabid animal. Regarding rabies prophylaxis, there was a significant difference between what respondents reported doing and what they recognized as recommended action. Eighteen percent of respondents (49/270) recommended PEP for suspected rabid animal bites, and 4% (12/270) would seek PEP if bitten by a bat (Table 2).

The risk of exposure to bats was over three times greater among people who reported entering a bat refuge (OR 3.43, 95% CI 1.9-6.2) and over two times greater among individuals who had spent more than five years living or working near bat refuges or habitats (OR 2.53, 95% CI 1.4-4.5) or for persons who reported an agricultural occupation (OR 2.8, 95% CI 1.13-6.94) (Table 2). Respondents reporting prior exposure to bats were almost seven times more likely to report PEP than persons with no prior exposure to bats.

There were no differences in the dwelling conditions among the exposed and non-exposed households (Table 3).

**Table 3:** **Risk factors for exposure to bats among households near a bat refuge in Guatemala**

**Variables**	**Exposed to bats (N = 77)**	**Non-exposed to bats (N = 193)**	**Total (N = 270)**	**P-value**	**Odds ratio (95% CI)**
	**n (%)**	**n (%)**	**n (%)**		
More than 5 people living in household	35(45)	91(47)	126(47)	0.89	0.93(0.53-1.64)
Lived in the house less than a year	3(4)	13(7)	16(6)	0.56	0.56(0.09-2.13)
Windows/doors prevent bat entry	53(69)	128(66)	181(67)	0.77	1.12(0.61-2.07)
Pets or livestock	69(90)	155(80)	224 (83)	0.07	2.10(0.90-5.51)
Pets or livestock bitten by bats	18(23)	30(16)	48(18)	0.16	1.65(0.80-3.33)
≥1 pet/livestock vaccinated against rabies	49(64)	105(54)	154(57)	0.18	1.46(0;82–2.63)
Mosquito net used to prevent bat entry	12(16)	18(9)	30(11)	0.19	1.79(0.74-4.18)
Measures taken to avoid animals being bitten by bats	28(36)	65(34)	93(34)	0.67	1.12(0.62-2.01)

## Discussion

We surveyed two communities in southern Guatemala, located within 2 km of bat refuges, to determine the knowledge, attitudes and practices of persons towards bats and rabies. To our knowledge, this is the first study in Guatemala to address these issues, despite the risk of rabies represented by hematophagous bats. An overwhelming majority of respondents (90%) reported that they knew little or nothing about rabies. Results indicated a relatively high general awareness of rabies transmission mechanisms and disease severity, but only dog bites were perceived as a source of RABV transmission. In contrast, the risk of RABV transmitted by bat bites was rarely known, with only 10% of respondents identifying bats as a potential source of rabies and 14% of respondents failing to say they would take any specific action if bitten or scratched by a bat. Cats and wild mammals were infrequently recognized as possible sources of rabies. In a similar study among persons at risk of bat exposures in Thailand, a history of bat exposure (bite or scratch) was reported by 27% of respondents, the majority of survey respondents (54%) reported having little or no knowledge about rabies, and only 10% of participants named bats as a source of rabies whereas dogs were identified as a source by 76% of respondents [25].

Our observations differ from a similar KAP survey in Peru, in which households that reported pets or livestock bitten by bats were also more likely to have humans bitten by bats, and also in the observation of greater exposure risk among males [26]. Since we did not interview any persons less than 18 years of age, we were not able to determine differences in the risk of bat exposure between children and adults.

The finding of more females exposed than males could be caused by a bias since 73% of respondents were females it is possible to miss exposed males. This phenomenon was due to the majority of men in these communities are engaged in the crop of sugar cane or in coffee plantations, and were working in the field all day. The attempt to recruit more men after work hours was not successful.

In our study, while most of the respondents lacked knowledge regarding the basic measures to take if they were bitten by a bat or a suspected rabid animal, some reported the use of traditional or folk remedies. Non-exposed participants using such folk remedies would also seek PEP, indicating acceptability of proper prevention measures in coexistence with their local practices. Although only a tenth of the participants would seek PEP following a bat bite, around 60% would seek medical assistance, and this action may increase the probability of receiving PEP when it is available in the health care institution visited.

In Guatemala, the public health care system plays an essential role in rabies prevention. Level 1 and 2 health centres have a role in passive and active surveillance by collecting animal samples and delivering them to the health areas, from where they are sent to the nearest laboratory for testing. Level 2 centres provide canine rabies vaccinations free of charge throughout the year. Level 2 and 3 centres are able to administer human PEP following exposure to suspect rabid animals.

Anyone bitten by a dog can go to their nearest health centre for PEP. If this is a level 1 centre, the person will be referred to an appropriate health centre if necessary. Health centres level 2 and 3 in theory carry a supply of human vaccine, which is administered without charge to the patient. Rabies Immune globulin is not available within the Guatemalan public health system [27]. Both communities in our survey lacked a level 2 health centre, but both could access a level 1 health centre within a 10 minute drive.

While 41 participants had been bitten by a bat, only 17 reported receiving PEP. To date, no human rabies cases associated with bats have been identified in Guatemala, although characterization of the RABV variants responsible for human and animal cases is not routinely performed in the country.

Rabies cases in livestock may be associated with bat RABV (especially where cases in dogs are rare), and occur mostly in northern Guatemala, where the cattle industry has a greater presence. Between 2005 and 2012, no cases of rabies in cattle were reported from the Department of Escuintla, and only one livestock case was reported from the Department of Santa Rosa, while 204 livestock cases were reported for the entire country [28]. There are no reports of any human rabies case in our study area, and this may indicate low RABV circulation in the bat population. This hypothesis is consistent with the results of an enhanced bat rabies surveillance study in Guatemala, which reported low prevalence (0.3%) of rabies virus neutralizing antibodies in bats from several communities including El Naranjo and El Jobo [29].

Our findings are relevant for the study and risk assessment of other bat pathogens besides rabies. In recent years, bats were sampled in several areas of Guatemala including the areas of our study, resulting in the report of high prevalence of *Bartonella sp.*infection in bats [30], the first-ever report of an influenza A virus infection in New World bats [31], as well as reports of polyomaviruses [32], hepaciviruses and pegiviruses from bats [33]. Furthermore, bats in Guatemala have been associated with several important infectious diseases. In the 1970s, researchers demonstrated antibodies against Venezuelan Equine Encephalitis Virus (VEE) in several bat species in Guatemala [23]. In a different study, neutralizing antibodies to VEE, vesicular stomatitis, eastern equine encephalitis, western equine encephalitis, St. Louis encephalitis, Tacaribe and Rio Bravo viruses were detected among bats in Guatemala [19]. In 2005, histoplasmosis, a fungus associated with bat droppings, was reported in tourists who visited bat caves in Guatemala [20]. More epidemiological studies are needed to better assess the risks associated with bat-related exposures, particularly in regions of the country where outbreaks of rabies and detection of other bat- associated pathogens have been documented, as questions remain regarding natural reservoirs and zoonotic potential for many of these pathogens.

There are some limitations to our study that should be noted. First, our findings may have been subject to recall bias since we ask for any exposure in time life. A person who was bitten by a bat more probably could remember the event more frequently than a person who handled it. Second, since most of the respondent enrolled in the survey bellows to the less of 46 years age group, our observation of less exposure risk among respondents in younger age group probably was result of a bias in the age group of the enrolled persons. The amount of not exposed persons in the range of less of 46 years (the media of the exposed person age) represents the 67% (182/270) of the whole respondents, and with 60% of respondents not exposed (193/270), the likelihood of being a non-exposed person in that age range is very high, so this result should be interpreted with caution.

## Conclusions

Although the decreasing incidence of human rabies in Guatemala [34] suggests the effectiveness of past and present rabies prevention through vaccination and education efforts targeting human-dog interactions, our findings demonstrate a need to raise public awareness of the potential risk of rabies associated with bat exposures, and targeting communities where bats roost in close proximity to human populations. Education at the community level is an important strategy in the prevention of human rabies [35]. School-based programs should include primary level students to ensure that the message reaches those who do not progress beyond this level of schooling. It is also important to emphasize the value of exposure avoidance and to promote wound cleansing and healthcare utilization following bat bites or scratches.

It would be useful to understand under what circumstances the handling bats with unprotected skin occurred – i.e. bats trapped on fences, located in residences, hunting etc. bat exposures are most common when humans interacted with trapped or sick bats. Trapped or sick bats are more likely to carry a lyssavirus [36], this should be included in future surveys.

Because we surveyed only two communities in a rural part of southern Guatemala, our results may not be representative of the entire country, but provide valuable information for initiating programs to increase awareness among at-risk populations regarding the potential risk of bat exposures, and to communicate the availability of effective PEP in case of animal bite exposures. Future studies should target northern areas of Guatemala where the burden of rabies in livestock (and bats) is suspected to be high, to ascertain whether residents in those regions are more sensitized to the health risks associated with bat bites and to deliver relevant educational messages to these communities. This study serves as a baseline for future investigations of the human-animal interface in Guatemala, and to perform comparative analyses between different regions of the country to understand spatiotemporal variation in bat exposures as they relate to risk of rabies and other diseases.

## Abbreviations

PreP: Pre exposure immunization
PEP: Post exposure prophylaxis
RABV: Rabies virus
MSPAS: Ministry of Public Health and Social Assistance
UVG: Universidad del Valle de Guatemala
CDC: Centers for Diseases Control and Prevention
OR: Odds ratio
CI: Confidence interval
KAP: Knowledge, attitudes and practices
VEE: Venezuelan Equine Encephalitis
